# Mediating Effects of Information Access on Internet Use and Multidimensional Health Among Middle-Aged and Older Adults: Nationwide Cross-Sectional Study

**DOI:** 10.2196/49688

**Published:** 2024-09-09

**Authors:** Liping Fu, Caiping Liu, Yongqing Dong, Xiaodong Ma, Quanling Cai, Dongli Li, Kaisheng Di

**Affiliations:** 1 College of Management and Economics Tianjin University Tianjin China; 2 College of Politics and Public Administration Qinghai Minzu University Xining China; 3 College of Chunming Hainan University Haikou China

**Keywords:** internet use, health, middle-aged and older adults, information access, mediation analysis

## Abstract

**Background:**

With the exacerbation of population aging, the health issues of middle-aged and older adults have increasingly become a focus of attention. The widespread use of the internet has created conditions for promoting the health of this demographic. However, little is known about the effects of information access in promoting the relationship between internet use and the health of middle-aged and older adults.

**Objective:**

This study aims to examine the relationship between internet use and multidimensional health in middle-aged and older adults, as well as the mediating effect of information access. Moreover, this study will explore the relationship between other dimensions of internet use (purposes and frequency) and health.

**Methods:**

Data were sourced from the China General Social Survey conducted in 2018. Health outcomes, including self-rated, physical, and mental health, were assessed using the 5-level self-rated health scale, the 5-level basic activities of daily living scale, and the 5-level depression scale, respectively. The ordinal logistic regression model was used to examine the relationship between internet use and health among middle-aged and older adults. Additionally, the Karlson-Holm-Breen decomposition method was used to examine the mediation effect of information access. To address endogeneity issues, the two-stage least squares approach was applied.

**Results:**

In our sample, nearly half (n=3036, 46.3%) of the respondents use the internet. Regression analyses revealed that internet use was positively associated with self-rated health (odds ratio [OR] 1.55, 95% CI 1.39-1.74; *P*<.001), physical health (OR 1.39, 95% CI 1.25-1.56; *P*<.001), and mental health (OR 1.33, 95% CI 1.19-1.49; *P*<.001) of middle-aged and older adults. Various dimensions of internet use positively contribute to health. In addition, information access significantly mediated the relationship between internet use and self-rated health (β=.28, 95% CI 0.23-0.32), physical health (β=.40, 95% CI 0.35-0.45), and mental health (β=.16, 95% CI 0.11-0.20). Furthermore, there were significant differences in the relationship between internet use and health among advantaged and disadvantaged groups.

**Conclusions:**

The study showed that different dimensions of internet use are associated with better self-rated health, better physical health, and better mental health in middle-aged and older adults. Information access mediates the relationship between internet use and health. This result emphasizes the significance of promoting internet access as a means to enhance the health of middle-aged and older adults in China.

## Introduction

### Background

With the extension of human life expectancy and the decline in birth rates, the global trend of population aging is becoming increasingly pronounced [1]. This phenomenon poses significant challenges to health care and social security systems, making the promotion of healthy aging crucial for socioeconomic development and realizing global health governance [2]. Health is the foundation for enhancing life expectancy, improving quality of life, and achieving healthy aging [3].

In today’s world, with the advancement of information technology, the internet is integrating into various aspects of society, profoundly changing people’s ways of production and living. According to Digital 2022: Global Digital Reports, as of January 2022, global internet users have reached 4.95 billion, with an internet penetration rate of 62.5% [4]. Meanwhile, as of December 2022, China’s internet users have surpassed 1.067 billion, including 153 million older people internet users [5]. The rapid spread and development of the internet have had a significant influence on the field of health. For instance, it enables access to health information [6], intervenes in health behaviors [7], monitors public health [8], formulates health policies [9], and provides medical services [10].

Evidence is mixed on the influence of internet use on health in middle-aged and older adults. One perspective suggests that the internet positively influences health and is considered an effective tool for improving health status [11-13]. Evidence indicates that middle-aged and older adults view the internet as a communication platform for expressing emotions and maintaining social relationships [14]. The internet has established novel social connections, expanding the external social networks of older individuals and helping them regain social opportunities [15]. Furthermore, the internet has strengthened intergenerational family bonds [16]. It has substituted face-to-face communication methods, reconstructing a web-based family space for older individuals. Adult children can provide telecare services for older adults, thereby partially fulfilling the expectations of intimacy that older individuals have toward their children [17]. In summary, the internet has created a new social space for older adults not limited by physical boundaries, significantly enhancing their overall physical and mental health and positively influencing intergenerational relationships [18,19]. In addition, the internet’s web-based consultation and telemedicine have enhanced the accessibility of health care services for middle-aged and older adults [20,21]. However, another perspective suggests that internet use may adversely affect health [22-24]. Research from the Stanford Institute for the Quantitative Study of Society and the Kennedy School of Government has revealed that internet use does not reduce social isolation [25,26]. It may even be associated with a higher risk of severe social isolation in certain situations [27]. Similarly, some studies indicate that excessive internet use can lead to internet addiction, causing distress to individuals’ psychological, social, and occupational well-being [28]. Other research also suggests that excessive internet use can lead to reduced rest time, which may harm the health of middle-aged and older adults [29].

A possible mediator between internet use and health association is information access. The health and wellness model supports this viewpoint, indicating that individuals with health consciousness can better understand their health status, including disease risks, preventative measures, and treatment options, through the acquisition of accurate health information [30,31]. This contributes to heightened health awareness and encourages people to engage in proactive health behaviors such as regular checkups, healthy eating, and exercise [32]. Additionally, information retrieval can provide psychological support and coping strategies, aiding in managing stress and anxiety, and thereby improving mental health [33,34]. In summary, information access plays a key role in maintaining and enhancing health conditions, facilitating individuals in effectively managing and preserving their health [35,36]. However, Chinese scholars have not yet given sufficient consideration to interpreting the influence of internet use on the health of middle-aged and older adults from an information perspective. Research on this issue is not only important for understanding the factors influencing the health of middle-aged and older adults but also for gaining insights into the socioeconomic consequences of internet use.

Similarly, the literature debates the relationship between internet use and social inequality. Some studies conclude that during the early and middle stages of internet adoption, advantaged groups with higher income, higher education, and more excellent technological proficiency are likelier to access the internet and enjoy its benefits [37-39]. Conversely, disadvantaged groups faced information disadvantages due to limitations in cognitive abilities and lower digital literacy, contributing to the digital divide [40,41]. However, other studies have concluded that the widespread availability of the internet has lowered the barriers to entry, increasing the possibility for all social groups, especially disadvantaged groups, to access information resources and technology, thus promoting upward mobility among society’s marginalized populations [42,43]. According to the law of diminishing marginal utility, the benefits of internet use are limited in populations with substantial existing information resources. Instead, the benefits of the internet may be more concentrated among disadvantaged groups, leading to digital dividends [44,45]. So, whether the internet’s effect on health brings about a digital divide or digital dividend and whether it narrows health inequalities between advantaged and disadvantaged groups or widens disparities among different populations needs to be empirically tested in this study.

There are several significant research gaps in the existing literature. First, although there is increasing interest in the potential influence of the internet on health [46], there is still a relative shortage of in-depth research explicitly targeting middle-aged and older adults in China. More comprehensive studies are required to investigate the relationship between multidimensional internet use and multidimensional health. Furthermore, limited existing literature categorizes middle-aged and older adults into advantaged and disadvantaged groups based on multiple dimensions within the same sample, and investigates the relationship between internet use and their health. In the diverse social context of China, it is essential to conduct in-depth research to investigate whether there are differences in the influence of internet use on the health of advantaged and disadvantaged groups. Such research can help us better understand the potential of the internet to reduce or widen health inequalities, thus providing a scientific basis for formulating more equitable and effective public health policies. In summary, there is significant room for further exploration in studying internet use and its relationship with health among middle-aged and older adults in China. More in-depth and comprehensive exploration is needed to address the current research gaps in this field.

### Objectives

Based on the analysis provided in the background section, this study aims to analyze the relationship between internet use and the multidimensional health of middle-aged and older adults using a nationally representative survey in China. Additionally, it investigates the relationship between the purposes and frequency of internet use and multidimensional health. Furthermore, this study extends the existing literature by examining the mediating effects of information access on the association between information use and multidimensional health. Moreover, the study will categorize middle-aged and older adults into advantaged and disadvantaged groups based on 4 criteria: income level, educational attainment, urban-rural classification, and region. Subsequently, this study will unveil potential heterogeneous influences for different subgroups within the advantaged and disadvantaged groups.

## Methods

### Data and Sample

**Figure 1:**
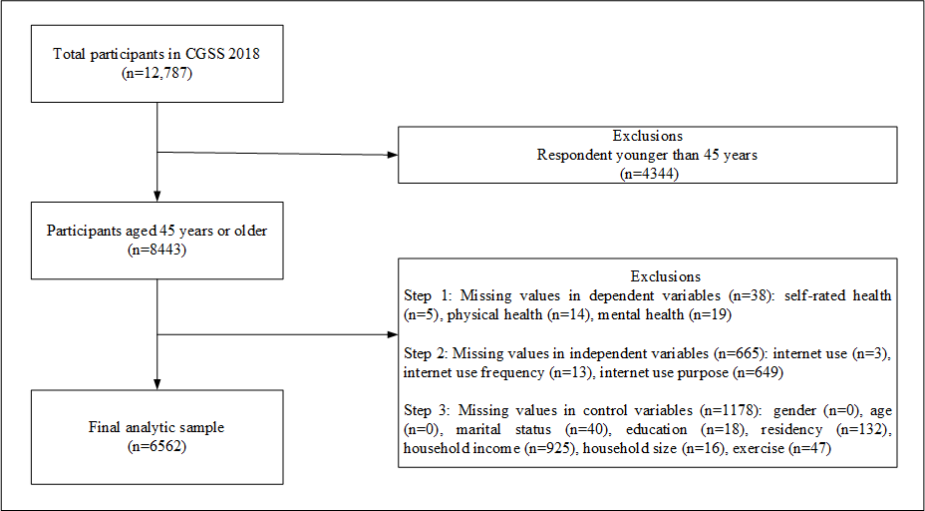
Participant screening flowchart. CGSS: China General Social Survey.

The China General Social Survey (CGSS) is China’s earliest, nationally representative, and comprehensive ongoing academic survey initiative [47]. The project uses a stratified, multistage probability proportional to size method to collect data, comprehensively gathering data at multiple levels including society, communities, households, and individuals. It covers 28 provinces, autonomous regions, and municipalities across China [48]. This study used nationwide survey data from CGSS2018. Following the age classification standards of the World Health Organization and considering the situation in China, individuals aged 45 years and older were defined as middle-aged and older adults [49]. Among the participants in CGSS2018, a total of 12,787 individuals were surveyed. After excluding 4344 individuals younger than 45 years, and additionally removing cases with missing data, a final valid sample of 6562 individuals was obtained. Figure 1 presents the specific data processing procedures.

To examine whether internet use widens the digital divide between the advantaged and disadvantaged groups or brings more information welfare to the disadvantaged group, this study classified middle-aged and older adults into advantaged and disadvantaged groups based on 4 criteria. The criteria include income level, educational attainment, urban-rural classification, and region. The disadvantaged group refers to middle-aged and older adults with income below the median, an education level at or below junior high school, residing in rural areas, or living in western regions of China. Conversely, those not meeting these criteria are categorized as the advantaged group. This differentiation between advantage and disadvantage is measured using objective criteria and has no discriminatory nature.

### Ethical Considerations

CGSS was a survey approved by the Ethical Review Committee of Renmin University of China. The survey data was also anonymous, and the answers were protected by privacy law. Each participant provided signed informed consent at the time of participation. There was no requirement for additional ethics approval for approved data users.

### Measures

#### Dependent Variable

Similar to prior research [50], health was measured using 3 indicators: self-rated health, physical health, and mental health.

Self-rated health, although simple, has been demonstrated to predict mortality and disability rates successfully [51]. Self-rated health (“How do you rate your current health?”) was assessed using a 5-point scale (1=very unhealthy, 2=relatively unhealthy, 3=average, 4=relatively healthy, and 5=very healthy). The higher the value, the healthier.

Physical health (“In the past 4 weeks, how frequently has your work or other daily activities been affected by health problems?”) was assessed using a 5-point scale (1=always, 2=often, 3=sometimes, 4=rarely, and 5=never), with higher values indicating better physical health.

Mental health (“How frequently have you felt symptoms of depression or sadness in the past 4 weeks?”) is assessed using a 5-point scale (1=always, 2=often, 3=sometimes, 4=rarely, and 5=never), with higher values indicating better mental health status.

#### Independent Variables

The core independent variable in this study is internet use. Respondents were asked whether they use the internet (yes=1 and no=0). Additionally, the CGSS questionnaire includes questions regarding internet use frequency (never=1, rarely=2, sometimes=3, often=4, and very frequently=5), with higher values indicating more frequent internet use. Moreover, the CGSS questionnaire includes questions about the purposes of internet use, such as web-based payment (yes=1 and no=0), web-based socializing (yes=1 and no=0), web-based learning (yes=1 and no=0), and web browsing (yes=1 and no=0).

#### Mediating Variable

The mediating variable is information access. Information access was measured using the question “Is the internet (including mobile internet) your main source of information?” with “yes” coded as 1 and “no” as 0.

#### Control Variables

According to the health capital demand theory and existing research literature [52,53], this study controlled for individual sociodemographic and economic variables. These variables include gender (male=1 and female=0), age (continuous variable), educational level (illiterate=1, primary school or below=2, junior high school=3, high school=4, and college or above=5), marital status (married=1 and unmarried=0), residence (rural=0 and urban=1), household annual income (continuous variable), household size (continuous variable), and whether to exercise (yes=1 and no=0).

#### Instrumental Variables

We used 2 instrumental variables to address endogeneity issues. The first instrumental variable is the mean of the frequency of internet use by other community residents (excluding the respondent’s internet use). The second instrumental variable is the broadband download rate of the respondents’ province. Table S1 in Multimedia Appendix 1 presents the definition and coding of variables.

### Statistical Analysis

All statistical analyses were conducted using Stata software (version 17.0; StataCorp LLC), with *P* values <.05 considered statistically significant (2-tailed).

We performed a descriptive analysis of the sample, summarizing continuous data using means and SDs, and presenting categorical data as frequencies and percentages. In the context of univariate analysis, we examined the relationship between internet use and health. The chi-square test was applied for categorical variables, while the Mann-Whitney *U* test was used for continuous variables.

Additionally, we used an ordinal logistic regression model to examine the odds ratios (ORs) and 95% CI for the association between internet use and health. Three models were constructed: model 1 included internet use as the independent variable and self-rated health, physical health, and mental health as the dependent variables, without any additional control variables. Model 2 incorporated sociodemographic variables such as gender, age, education, residency, marital status, and exercise. Model 3 introduced family variables, including family size and family income, on the basis of model 2. Models 2 and 3 both included provincial fixed effects to adjust for unobserved provincial-level factors.

To provide a comprehensive understanding of the research, we conducted the following analyses: first, we explored the relationship between internet use and health, the purposes of internet use and health, as well as the relationship between the frequency of internet use and health. All 3 sets of regressions used an ordinal logistic regression model. Second, we used the Karlson-Holm-Breen (KHB) decomposition method to examine whether information access mediated the relationship between internet use and health. Third, to address potential endogeneity problems, we introduced instrumental variable methods, specifically using a two-stage least squares regression. Fourth, middle-aged and older adults were categorized into advantaged and disadvantaged groups based on 4 criteria. Further analysis was conducted using grouped regression analysis for the advantaged and disadvantaged subgroups. Finally, it should be noted that all our regression analyses incorporated provincial fixed effects to control for provincial disparities.

## Results

### Descriptive Statistics

Table 1 summarizes the sociodemographic characteristics of the study participants (n=6562). From a demographic perspective, the average age of the participants was 61.41 (SD 10.69) years. The gender ratio between males and females (n=3392, 51.69% vs n=3170, 48.31%) was approximately balanced. Most participants (n=5342, 81.41%) had partners, while a minority (n=1220, 18.59%) were single. The educational level of the participants was relatively low (n=4875, 74.29%), with approximately three-quarters having yet to receive education beyond high school. Regarding geographical distribution, there were more participants from rural areas than urban areas (n=4067, 61.98% vs n=2495, 38.02%). In terms of family characteristics, the average household size was 2.55 (SD 1.33), indicating that at least two people lived together in each household, with few individuals living alone.

**Table 1:** Descriptive statistics of variables (n=6562).

Characteristics and variables		Total sample (n=6562)	Internet use (n=3036)	Internet nonuse (n=3526)	*P* value
**Self-rated health, n (%)**					<.001
	Very unhealthy	301 (4.59)	59 (1.94)	242 (6.86)	
	Relatively unhealthy	1372 (20.91)	391 (12.88)	981 (27.82)	
	Average	1719 (26.20)	802 (26.42)	917 (26.01)	
	Relatively healthy	2355 (35.89)	1282 (42.23)	1073 (30.43)	
	Very healthy	815 (12.41)	502 (16.53)	313 (8.88)	
**Physical health, n (%)**					<.001
	Very unhealthy	286 (4.36)	50 (1.65)	236 (6.69)	
	Relatively unhealthy	834 (12.71)	200 (6.59)	634 (17.98)	
	Average	1245 (18.97)	436 (14.36)	809 (22.94)	
	Relatively healthy	2060 (31.39)	1094 (36.03)	966 (27.40)	
	Very healthy	2137 (32.57)	1256 (41.37)	881 (24.99)	
**Mental health, n (%)**					<.001
	Very unhealthy	85 (1.30)	23 (0.76)	62 (1.76)	
	Relatively unhealthy	533 (8.12)	161 (5.30)	372 (10.55)	
	Average	1561 (23.79)	594 (19.57)	967 (27.43)	
	Relatively healthy	2495 (38.02)	1192 (39.26)	1303 (36.95)	
	Very healthy	1888 (28.77)	1066 (35.11)	822 (23.31)	
**Sex, n (%)**					.003
	Male	3392 (51.69)	1510 (49.74)	1882 (53.37)	
	Female	3170 (48.31)	1526 (50.26)	1644 (46.63)	
Age (years), mean (SD)		61.41 (10.69)	56.92 (9.17)	65.27 (10.39)	<.001
**Marital status, n (%)**					<.001
	Partnered	5342 (81.41)	2662 (87.68)	2680 (76.01)	
	Single	1220 (18.59)	374 (12.32)	846 (23.99)	
**Education, n (%)**					<.001
	Illiterate	1227 (18.70)	186 (6.13)	1.41 (29.52)	
	≤Primary school	1813 (27.63)	501 (16.50)	1312 (37.21)	
	Middle school	1835 (27.96)	1.38 (34.19)	797 (22.6)	
	High school	1150 (17.53)	834 (27.47)	316 (8.96)	
	≥College	537 (8.18)	477 (15.71)	60 (1.70)	
**Residency, n (%)**					<.001
	Urban	2495 (38.02)	1657 (54.58)	838 (23.77)	
	Rural	4067 (61.98)	1379 (45.42)	2688 (76.23)	
**Exercise, n (%)**					<.001
	Yes	3122 (47.58)	1811 (59.65)	1311 (37.18)	
	No	3440 (52.42)	1225 (40.35)	2215 (62.82)	
Family size, mean (SD)		2.55 (1.33)	2.58 (1.24)	2.53 (1.39)	<.001
Family income, mean (SD)		10.10 (2.19)	10.80 (1.66)	9.49 (2.40)	<.001

Regarding health status, among the participants, 35.89% (n=2355) considered their self-rated health relatively healthy, and 12.41% (n=815) considered it to be very healthy. In terms of physical health, 31.39% (n=2060) reported themselves as relatively healthy, while 32.57% (n=2137) reported themselves as very healthy. Concerning mental health, 38.02% (n=2495) rated their mental health as relatively healthy, while 28.77% (n=1888) rated it as very healthy. Overall, the health status of middle-aged and older adults was relatively good.

Among participants who rated their self-rated health as unhealthy (including very unhealthy and relatively unhealthy), 450 (6.86%) individuals had used the internet, while 1223 (18.64%) individuals had not used the internet. In the case of participants who assessed their physical health as unhealthy (including very unhealthy and relatively unhealthy), 250 (3.80%) individuals had used the internet, while 870 (13.26%) individuals had not used the internet. Likewise, among participants who considered their mental health as unhealthy (including very unhealthy and relatively unhealthy), 184 (2.80%) individuals had used the internet, while 434 (6.61%) individuals had not used the internet. The descriptive statistics showed that middle-aged and older adults with poor self-rated, physical, and mental health are more likely to not use the internet (*P*<.001). Additionally, females (*P*<.001), older individuals (*P*<.001), those with less than a high school education (*P*<.001), and those without a partner (*P*<.001) are more inclined not to use the internet. Furthermore, individuals with lower household income (*P*<.001), smaller household size (*P*<.001), and those living in rural areas (*P*<.001) were also more likely to refrain from using the internet.

Table 2 displays the frequency and purpose of internet use. In the middle-aged and older adult population, internet use frequency is relatively low. Over half of the individuals (n=3526, 53.73%) have never used the internet, while only 15.33% (n=1006) use it often, and 13.2% (n=866) use it very frequently. A total of 27.52% (n=1806) of middle-aged and older adults use the internet for information access, 41.31% (n=2711) for web-based learning, 44.93% (n=2948) for web-based socializing, 17.16% (n=1126) for web-based payment, and 44.38% (n=2912) for web browsing.

**Table 2:** Internet use frequency and purpose by respondents in the sample (n=6562).

Variable		Participants, n (%)
**Internet use frequency**		
	Never	3526 (53.73)
	Rarely	542 (8.26)
	Sometime	622 (9.48)
	Often	1006 (15.33)
	Very frequently	866 (13.20)
**Internet use purpose (reference: yes)**		
	Information access	1806 (27.52)
	Web-based learning	2711 (41.31)
	Web-based socializing	2948 (44.93)
	Web-based payment	1126 (17.16)
	Web browsing	2912 (44.38)

### Internet Use and Multidimensional Health

Table 3 presents the results of the regression analysis, illustrating the relationship between internet use and multidimensional health. Internet use demonstrated a significant positive association with self-rated health, physical health, and mental health. The findings indicated that middle-aged and older adults who used the internet were more likely to report good self-rated health compared to nonusers (OR 1.55, 95% CI 1.39-1.74; *P*<.001). Additionally, individuals who used the internet had significantly higher odds of better physical health than nonusers (OR 1.39, 95% CI 1.25-1.56; *P*<.001). Moreover, individuals who used the internet were more likely to have positive mental health compared to nonusers (OR 1.33, 95% CI 1.19-1.49; *P*<.001). This section presents only the results of model 3, while the regression results of models 1 and 2 can be found in Table S2 in Multimedia Appendix 1.

**Table 3:** Regression results for health among study participants (n=6562).

Variable		Self-rated health, OR^a^ (95% CI)	*P* value	Physical health, OR (95% CI)	*P* value	Mental health, OR (95% CI)	*P* value
**Internet use**							
	Yes (reference: no)	1.55 (1.39-1.74)	<.001	1.39 (1.25-1.56)	<.001	1.33 (1.19-1.49)	<.001
**Sex**							
	Male (reference: female)	1.28 (1.17-1.41)	<.001	1.26 (1.14-1.38)	<.001	1.29 (1.18-1.42)	<.001
Age		0.98 (0.97-0.98)	<.001	0.98 (0.97-0.98)	<.001	1.01 (1.00-1.01)	.009
**Marital status**							
	Married (reference: unmarried)	1.07 (0.95-1.21)	.27	1.16 (1.02-1.31)	.02	1.26 (1.11-1.43)	<.001
**Education**							
	≤Primary school (reference: illiterate)	1.06 (0.93-1.22)	.38	1.05 (0.92-1.20)	<.001	1.08 (0.94-1.23)	.29
	Middle school (reference: illiterate)	1.18 (1.01-1.37)	.04	1.35 (1.16-1.57)	.51	1.28 (1.11-1.49)	.001
	High school (reference: illiterate)	1.37 (1.15-1.63)	<.001	1.56 (1.31-1.86)	<.001	1.34 (1.12-1.60)	.001
	≥College (reference: illiterate)	1.37 (1.10-1.71)	.006	1.60 (1.28-2.01)	<.001	1.21 (0.97-1.52)	.10
**Exercise**							
	Yes (reference: no)	1.57 (1.43-1.73)	<.001	1.52 (1.38-1.68)	<.001	1.36 (1.24-1.50)	<.001
**Residency**							
	Urban (reference: rural)	0.85 (0.75-0.96)	.009	1.02 (0.90-1.15)	.80	1.11 (0.98-1.25)	.09
	Family size	1.02 (0.99-1.06)	.24	1.03 (0.99-1.07)	.14	1.02 (0.98-1.06)	.28
	Family income	1.09 (1.06-1.11)	<.001	1.06 (1.04-1.09)	<.001	1.05 (0.02-1.07)	<.001

^a^OR: odds ratio.

### Frequency and Purpose of Internet Use and Health

Table 4 displays the associations between the frequency and purpose of internet use and health. From the analysis results, it becomes evident that the higher the frequency of internet use among middle-aged and older adults, the better their self-rated health (very frequently: OR 1.48, 95% CI 1.26-1.74; *P*<.001), physical health (very frequently: OR 1.76, 95% CI 1.48-2.08; *P*<.001), and mental health (very frequently: OR 1.52, 95% CI 1.28-1.79; *P*<.001) tend to be. Regarding the purposes of internet use, except for web-based payment (OR 1.08, 95% CI 0.94-1.24; *P*=.30), which had no relationship on the physical health of middle-aged and older adults, all other purposes of internet use showed significant associations with health. Compared to the control group, middle-aged and older adults who used the internet showed improvements in self-rated, physical, and mental health.

**Table 4:** Associations between health and internet use frequency and purpose (n=6562).

Independent variables: internet use		Self-rated health, OR^a^ (95% CI)	*P* value	Physical health, OR (95% CI)	*P* value	Mental health, OR (95% CI)	*P* value
**Frequency (reference: never)**							
	Rarely	1.64 (1.38-1.94)	<.001	1.25 (1.05-1.47)	.01	1.27 (1.07-1.51)	.006
	Sometimes	1.41 (1.19-1.66)	<.001	1.28 (1.08-1.51)	<.004	1.20 (1.02-1.42)	.03
	Often	1.67 (1.44-1.94)	<.001	1.44 (1.23-1.67)	<.001	1.39 (1.19-1.62)	<.001
	Very frequently	1.48 (1.26-1.74)	<.001	1.76 (1.48-2.08)	<.001	1.52 (1.28-1.79)	<.001
**Purpose (reference: no)**							
	Web-based payment	1.24 (1.08-1.42)	.002	1.08 (0.94-1.24)	.30	1.22 (1.06-1.41)	.005
	Web-based learning	1.49 (1.33-1.67)	<.001	1.41 (1.25-1.58)	<.001	1.29 (1.15-1.45)	<.001
	Web-based socializing	1.51 (1.34-1.70)	<.001	1.58 (1.41-1.78)	<.001	1.48 (1.3-1.66)	<.001
	Web browsing	1.40 (1.25-1.57)	<.001	1.39 (1.24-1.55)	<.001	1.29 (1.15-1.45)	<.001

^a^OR: odds ratio.

### Mediation Effect of Information Access

Table 5 reports the mediation effect of information access using the KHB decomposition method. The results revealed that information access had a significant mediating effect (β=.28, 95% CI 0.23-0.32) on the relationship between internet use and self-rated health. Additionally, information access also had a significant mediating effect (β=.40, 95% CI 0.35-0.45) on the relationship between internet use and physical health. Moreover, the KHB results showed that the indirect effect of information access on internet use and mental health was significant (β=.16, 95% CI 0.11-0.20). Therefore, the mediating effect of information access on internet use and each dimension of health among middle-aged and older adults was significant.

**Table 5:** Mediation of the relationship between middle-aged and older adults’ health and internet use by information access (n=6562).

Mediation by information access	Self-rated health, β (95% CI)	*P* value	Physical health, β (95% CI)	*P* value	Mental health, β (95% CI)	*P* value
Total effect	.52 (0.47-0.57)	<.001	.63 (0.58-0.68)	<.001	.33 (0.29-0.38)	<.001
Direct effect	.24 (0.18-0.31)	<.001	.23 (0.16-0.30)	<.001	.18 (0.12-0.24)	<.001
Indirect effect	.28 (0.23-0.32)	<.001	.40 (0.35-0.45)	<.001	.16 (0.11-0.20)	<.001

### Treatment: Instrumental Variables Approach

Internet use as a form of individual decision-making may lead to endogeneity issues due to reverse causality or omitted variables. While significant efforts have been made in this paper to carefully select control variables to mitigate omitted variable issues, endogeneity concerns may still exist. To address this issue, the study sought instrumental variables for the internet use of middle-aged and older adults to mitigate potential bias and nonconsistency resulting from endogeneity. Ultimately, 2 instrumental variables were chosen, and a two-stage estimation was performed using the product of these two instrumental variables as instruments. The first instrumental variable is the mean internet use frequency among residents in the respondent’s community (excluding the respondent’s internet use). The second instrumental variable is the broadband download rate of the province. Average bandwidth data for each province is sourced from the 19th edition of the “China Broadband Speed Report,” published by the Broadband Development Alliance.

Table S3 in Multimedia Appendix 1 displays the two-stage least squares regression. In the first-stage regression of the endogenous variables, the instrumental variables were statistically significant at the 1% significance level. The results from the second stage of the regression still suggested that individuals who used the internet had a higher likelihood of better self-rated health (β=.31, 95% CI 0.03-0.59; *P*=.03, better physical health (β=.55, 95% CI 0.25-0.86; *P*<.001), and better mental health (β=.33, 95% CI 0.08-0.59; *P*=.01) compared to nonusers. This suggested that the conclusions drawn earlier remained robust.

### Heterogeneity Analysis

In existing research [41,45], some scholars argue that the internet may create a digital divide between advantaged and disadvantaged groups, while others believe it may bring about digital dividends. To address this controversy, this study categorizes middle-aged and older adults into advantaged and disadvantaged groups with 4 criteria: income level, educational attainment, urban-rural classification, and region.

Table S4 in Multimedia Appendix 1 demonstrates that internet use had a significant facilitative effect on self-rated health (urban: OR 1.37, 95% CI 1.34-1.65; *P*=.001 vs rural: OR 1.67, 95% CI 1.45-1.93; *P*<.001), mental health (urban: OR 1.20, 95% CI 0.99-1.45; *P*=.06 vs rural: OR 1.40, 95% CI 1.22-1.62; *P*<.001) for both rural and urban samples, as well as physical health of the rural group (OR 1.57, 95% CI 1.36-1.81; *P*<.001). However, it had no significant effect on the physical health of the urban group (OR 1.12, 95% CI 0.93-1.36; *P*=.24). Therefore, for those in rural areas, the health effects of internet use were much larger.

Table S5 in Multimedia Appendix 1 reveals that the association between internet use and self-rated health (eastern: OR 1.45, 95% CI 1.24-1.69; *P*<.001 vs western: OR 2.03, 95% CI 1.60-2.57; *P*<.001), physical health (eastern: OR 1.21, 95% CI 1.03-1.41; *P*=.02 vs western: OR 2.14, 95% CI 1.69-2.71; *P*<.001), and mental health (eastern: OR 1.40, 95% CI 1.20-1.64; *P*<.001 vs western: OR 1.29, 95% CI 1.02-1.63; *P*=.04), for both western and eastern region samples were also significantly positive. However, for those in western areas, the effect was much larger.

Table S6 in Multimedia Appendix 1 displays that the association between internet use and self-rated health (high-income: OR 1.37, 95% CI 1.17-1.61; *P*<.001 vs low-income: OR 1.74, 95% CI 1.47-2.05; *P*<.001), physical health (high-income: OR 1.17, 95% CI 1.00-1.38; *P*=.05 vs low-income: OR 1.61, 95% CI 1.37-1.90; *P*<.001), and mental health (high-income: OR 1.16, 95% CI 0.98-1.36; *P*=.08 vs low-income: OR 1.49, 95% CI 1.26-1.76; *P*<.001) for both high-income and low-income samples were also significantly positive. However, for those in the low-income group, the effect was much larger.

Finally, as indicated in Table S7 in Multimedia Appendix 1, the association between internet use and self-rated health (high education: OR 1.56, 95% CI 1.34-1.80; *P*<.001 vs low education: OR 1.61, 95% CI 1.36-1.92; *P*<.001), physical health (high education: OR 1.49, 95% CI 1.28-1.73; *P*<.001 vs low-education: OR 1.29, 95% CI 1.09-1.53; *P*=.004), and mental health (high education: OR 1.35, 95% CI 1.17-1.57; *P*<.001 vs low education: OR 1.27, 95% CI 1.07-1.52; *P*=.007) for both high education and low-education samples were also significantly positive.

## Discussion

### Principal Findings

This study used data from China’s first nationwide publicly available database CGSS to examine the relationship between internet use and multidimensional health in middle-aged and older adults. We initially examined the significant relationships between internet use and self-rated, physical, and mental health. Subsequently, we conducted further analyses to explore the links between the purposes and frequency of internet use and multidimensional health. Then, we investigated the partial mediating role of information access in the relationship between internet use and health. Finally, we explored whether there were significant differences in internet use and health among advantaged and disadvantaged groups.

Our research confirmed that internet use was currently influencing the daily lives of middle-aged and older adults: 46.3% (n=3036) of middle-aged and older adults use the internet in our sample. This indicated that over half of the older people in our sample had not yet accessed the internet and were unable to benefit from the advancements in digital technology. This was still a significant gap compared to the internet penetration rates among older people in other countries, which ranged from 76% to 82% [54,55]. The reasons behind this phenomenon are multifaceted. First, the availability of smart devices and services for older adults is not yet comprehensive [56], and their digital skills are generally inadequate [57], resulting in fundamental operational obstacles during their use [58]. Second, the ownership of internet access devices among older adults is lower than younger generations [59]. Third, older adults have weaker information processing abilities and lower discernment skills, making them more susceptible to false information and telecommunications fraud [60]. These factors make some older adults unwilling to use the internet.

In this study, it was found that internet use was positively associated with the health of middle-aged and older adults. This contribution addressed the ongoing debate in the literature [18,22], as other surveys presented contradictory results. This could be related to the results of other studies [61] that the internet enables people to access health-related knowledge, gradually develop healthy lifestyle habits, and improve overall health. The internet provides middle-aged and older adults access to more health resources and convenient methods for managing their health, which positively affects their overall health [62,63]. For instance, middle-aged and older adults can use health apps and digital tools for medication management, health data recording, and health trend tracking, facilitating better self-management of health-related issues [64]. Furthermore, the internet offers crucial support for mental health, allowing middle-aged and older adults to access emotional support and resources to address psychological concerns through digital therapy [65]. Additionally, individuals’ internet use is associated with better financial and health care decision-making [66,67]. People who use the internet may have more financial resources, which can contribute to their health [38].

We found that information access significantly mediated the relationship between internet use and each dimension of health. The internet provides middle-aged and older adults access to a wealth of health-related information, from disease prevention to treatment recommendations, to understand better and manage their health [68,69]. They can enhance their health knowledge through web-based resources and adopt healthier lifestyles such as regular exercise and balanced diets [70]. Furthermore, information access reduces uncertainty in medical decision-making as individuals consult health care professionals through web-based platforms to seek treatment advice [71]. In summary, the information access feature of the internet enhances middle-aged and older adults’ understanding and management of their health, granting them greater autonomy and control over their well-being, ultimately resulting in a positive influence on overall health [72].

This study also confirmed that there were significant differences in the relationship between internet use and the health of middle-aged and older adults among advantaged and disadvantaged groups. Specifically, for those in rural and western areas, the effect was much larger. As pointed out in previous research [73], China faces an imbalance in the allocation of health care resources among regions, with a concentration of high-quality medical resources in urban and eastern regions [74]. In contrast, rural and western regions have relatively limited health care resources, resulting in insufficient accessibility to health care services [75]. Therefore, middle-aged and older adults in rural and western regions are more likely to improve their health through internet use [76]. This study also indicated that the association between internet use and the health of middle-aged and older adults was significantly positive for both higher-income and lower-income samples. However, the effect was greater for those in the lower-income group. Low-income disadvantaged group often faces challenges in accessing health care services than advantaged groups [77]. However, the internet provides them with abundant health information resources, including web-based consultations and medical advice, which help alleviate health care access issues linked to economic constraints [78]. In summary, internet use brings more benefits to disadvantaged groups [79]. This conclusion provides essential insights into how to mitigate health inequalities among advantaged and disadvantaged groups.

### Contributions

This study makes significant contributions to the existing literature from 4 perspectives. First, unlike previous research that often examined the relationship between internet use and health from a single perspective, we adopt an innovative research approach. We categorize internet use into 3 dimensions (whether to use the internet, internet use frequency, and internet use purpose) to provide a multidimensional consideration of internet use. Second, the statistical analysis in existing studies often overlooks endogeneity issues and sample selection bias. This paper addresses these concerns by selecting broadband download rates and internet use frequency among community residents as instrumental variables to ensure the reliability of the research results. Third, our findings provide empirical evidence for the first time, demonstrating that information access serves as a crucial mediating variable in the relationship between internet use and the health of middle-aged and older adults. This not only supports the theoretical proposition that internet use improves the health status of middle-aged and older adults but also advocates for the promotion of active aging policies. Fourth, to our knowledge, limited literature categorizes middle-aged and older adults into advantaged and disadvantaged groups based on multiple criteria and investigates the relationship between internet use and multidimensional health. Our results emphasize that internet use tends to benefit disadvantaged groups more. These insights are of significant importance and provide evidence for narrowing the health and welfare gap between different population groups.

### Limitations

Certainly, our study acknowledges specific limitations. First, due to data constraints, we have to rely on the question “How do you rate your current physical health?” to measure physical health. This may not comprehensively measure their health status. In future research, we will select more comprehensive physical health indicators, such as activities of daily living and instrumental activities of daily living, to better reflect the health status of individuals. Second, given the complexity of the relationship between internet use and health, future research should consider longitudinal analysis to understand better the causal relationships and temporal dynamics. Nonetheless, our research offers valuable evidence for low-income countries seeking a deeper understanding of the relationship between internet use and health.

### Conclusions

In this study, it was found that internet use was positively associated with better self-rated health, physical health, and mental health after controlling for key determinants of health. Additionally, information access mediated the relationship between internet use and the health of middle-aged and older adults in China. Furthermore, there were significant differences in the relationship between internet use and health among advantaged and disadvantaged groups. This study suggests collaborative efforts between the government and the market to further enhance internet accessibility for the older people population in China. Additionally, society should strive to lower the technological and income barriers for middle-aged and older adults in using the internet so that more seniors can enjoy the digital dividends brought by the internet.

## Appendix

Multimedia Appendix 1 Additional tables for the study.

## Abbreviations

CGSS: China General Social Survey
KHB: Karlson-Holm-Breen
OR: odds ratio

## Data Availability

The data sets generated during or analyzed during this study are available in the China General Social Survey repository [80].
