# Supplementary material for: Mediating Effects of Information Access on Internet Use and Multidimensional Health Among Middle-Aged and Older Adults: Nationwide Cross-Sectional Study
Source: J Med Internet Res. 2024 Sep 9;26:e49688. doi: 10.2196/49688 (PMC11420587; doi:10.2196/49688)
Supplement: Multimedia Appendix 1 [file jmir_v26i1e49688_app1.docx]

**Supplemental Table S1.** Definition and coding of variables.

**Supplemental Table S2.** Regression results for health among study participants(n=6562).

**Supplemental Table S3.** The treatment of endogeneity: instrumental variable model (n=6562).

**Supplemental Table S4.** Results for urban versus rural subgroups (n=6562).

**Supplemental Table S5.** Results for eastern versus western subgroups (n=6562).

**Supplemental Table S6.** Results for high-income versus low-income subgroups (n=6562).

**Supplemental Table S7.** Results for high-education versus low-education subgroups (n=6562).

**Table S1.** Definition and coding of variables.

| **Variables** | **Definition** |
| --- | --- |
| **Dependent variables** | |
| Self-rated health | Very unhealthy=1; Relatively unhealthy=2; Average=3; Healthy=4; Very healthy=5 |
| Physical health | Always=1; Often=2; Sometimes=3; Rarely=4; Never=5 |
| Mental health | Always=1; Often=2; Sometimes=3; Rarely=4; Never=5 |
| **Independent variable** | |
| Internet use | No = 0; Yes = 1 |
| **Control variables** |  |
| Gender | Female = 0; Male = 1 |
| Age | Continuous variables |
| Marriage | Unmarried = 0; Married = 1; |
| Education level | Illiterate =1; primary school or below =2; junior high school =3; high school =4; college or above =5 |
| Residency | Rural = 0; Urban = 1 |
| Exercise | No = 0; Yes = 1 |
| Family size | Continuous variables |
| Family income | Continuous variables |
| **Other variables** |  |
| **Other variables of internet ue** |  |
| Internet use frequency | Never=1; Rarely=2; Sometimes=3; Often=4; Very frequently=5 |
| **Purpose of internet use** |  |
| Information access | No = 0; Yes = 1 |
| Online learning | No = 0; Yes = 1 |
| Online payments | No = 0; Yes = 1 |
| Online socializing | No = 0; Yes = 1 |
| Web browsing | No = 0; Yes = 1 |
| **Instrumental Variable** | |
| Community Internet use frequency | Continuous variables (The mean of the frequency of Internet use by other community residents,excluding the respondent’s internet use) |
| Broadband download rate | Continuous variables (The broadband download rate of the respondents’ province) |

Note: In the questionnaire, the response options for physical health were as follows: Always = 1, Often = 2, Sometimes = 3, Rarely = 4, Never = 5. To make it easier for readers to understand, we converted these options in the analysis as follows: Always corresponds to "Very unhealthy," Often to "Relatively unhealthy," Sometimes to "Average," Rarely to "Healthy," and Never to "Very healthy." In other words, a higher score indicates a better physical health. The same approach was applied to mental health.

| **Model** | **Model 1^d^** | | |  | **Model 2^e^** | | |  | **Model 3^f^** | | |
| --- | --- | --- | --- | --- | --- | --- | --- | --- | --- | --- | --- |
| Variable | Self-rated health | Physical health | Mental health |  | Self-rated health | Physical health | Mental health |  | Self-rated health | Physical health | Mental health |
|  | OR(95% CI) | OR(95% CI) | OR (95% CI) |  | OR(95% CI) | OR(95% CI) | OR(95% CI) |  | OR (95% CI) | OR (95% CI) | OR(95% CI) |
| **Internet use** |  |  |  |  |  |  |  |  |  |  |  |
| Yes (ref: no) | 2.40^c^(2.19-2.62) | 2.64^c^(2.41-2.89) | 1.87^c^(1.71-2.04) |  | 1.64^c^(1.47-1.83) | 1.51^c^(1.35-1.69) | 1.43^c^(1.28-1.60) |  | 1.55^c^(1.39-1.74) | 1.39^c^(1.25-1.56) | 1.33^c^(1.19-1.49) |
| **Gender** |  |  |  |  |  |  |  |  |  |  |  |
| Male (re: female) | N/A^g^ | N/A | N/A |  | 1.28^c^(1.17-1.40) | 1.20^c^(1.10-1.32) | 1.24^c^(1.13-1.36) |  | 1.28^c^(1.17-1.41) | 1.26^c^(1.14-1.38) | 1.29^c^(1.18-1.42) |
| Age | N/A | N/A | N/A |  | 0.98^c^(0.97-0.98) | 0.98^c^(0.98-0.99) | 1.01^c^(1.01-1.02) |  | 0.98^c^(0.97-0.98) | 0.98^c^(0.97-0.98) | 1.01^b^(1.00-1.01) |
| **Marital status** |  |  |  |  |  |  |  |  |  |  |  |
| Married (ref: unmarried) | N/A | N/A | N/A |  | 1.13^a^(1.01-1.28) | 1.21^b^(1.08-1.37) | 1.32^c^(1.17-1.48) |  | 1.07(0.95-1.21) | 1.16^a^(1.02-1.31) | 1.26^c^(1.11-1.43) |
| **Education** |  |  |  |  |  |  |  |  |  |  |  |
| ≤Primary school (ref: illiterate) | N/A | N/A | N/A |  | 1.05(0.92-1.21) | 1.11(0.97-1.27) | 1.13(0.99-1.29) |  | 1.06 (0.93-1.22) | 1.05(0.92-1.20) | 1.08(0.94-1.23) |
| Middle school (ref: illiterate) | N/A | N/A | N/A |  | 1.20^a^(1.04-1.39) | 1.57^c^(1.36-1.81) | 1.45^c^(1.26-1.68) |  | 1.18^a^(1.01-1.37) | 1.35^c^(1.16-1.57) | 1.28^b^(1.11-1.49) |
| High school (ref: illiterate) | N/A | N/A | N/A |  | 1.38^c^(1.17-1.64) | 1.83^c^(1.55-2.17) | 1.54^c^(1.30-1.83) |  | 1.37^c^(1.15-1.63) | 1.56^c^(1.31-1.86) | 1.34^b^(1.12-1.60) |
| ≥College (ref: illiterate) | N/A | N/A | N/A |  | 1.47^c^(1.19-1.83) | 2.07^c^(1.66-2.58) | 1.51^c^(1.21-1.87) |  | 1.37^b^(1.10-1.71) | 1.60^c^(1.28-2.01) | 1.21(0.97-1.52) |
| **Exercise** |  |  |  |  |  |  |  |  |  |  |  |
| Yes (ref: no) | N/A | N/A | N/A |  | 1.61^c^(1.47-1.77) | 1.57^c^(1.43-1.72) | 1.40^c^(1.27-1.54) |  | 1.57^c^(1.43-1.73) | 1.52^c^(1.38-1.68) | 1.36^c^(1.24-1.50) |
| **Residency** |  |  |  |  |  |  |  |  |  |  |  |
| Urban (ref: rural) | N/A | N/A | N/A |  | 0.89^a^(0.79-0.99) | 1.26^c^(1.13-1.41) | 1.39^c^(1.25-1.56) |  | 0.85^b^(0.75-0.96) | 1.02(0.90-1.15) | 1.11(0.98-1.25) |
| Family size | N/A | N/A | N/A |  | N/A | N/A | N/A |  | 1.02(0.99-1.06) | 1.03(0.99-1.07) | 1.02(0.98-1.06) |
| Family income | N/A | N/A | N/A |  | N/A | N/A | N/A |  | 1.09^c^(1.06-1.11) | 1.06^c^(1.04-1.09) | 1.05^c^(1.02-1.07) |

**Table S2.** Regression results for health among study participants(n=6562).

^a^ *P*<.05, ^b^ *P*<.01, ^c^ *P*<.001

**^d^**Model 1 was a univariate model of internet use.

^e^Model 2 was adjusted for socioeconomic factors, such as gender, age, education, residency, marital status, and exercise.

^f^Model 3 was the final parsimonious model, further adjusted for family factors, including family size and family income, and also incorporated provincial fixed effects.

^g^N/A: not applicable.

**Table S3.** The treatment of endogeneity: instrumental variable model (n=6562).

|  | Results of the first stage regression | |  | Results of the second-stage regression | | | | | |
| --- | --- | --- | --- | --- | --- | --- | --- | --- | --- |
|  | Internet use |  |  | Self-rated health |  | Physical health |  | Mental health |  |
|  | B (95% CI) | *P* value |  | B (95% CI) | *P* value | B (95% CI) | *P* value | B (95% CI) | *P* value |
| Instrumental variable | 0.01(0.002-0.012) | <.001 |  | N/A |  | N/A |  | N/A |  |
| Internet use | N/A^a^ |  |  | 0.31(0.03-0.59) | .028 | 0.55(0.25-0.86) | <.001 | 0.33(0.08-0.59) | .010 |
| Control variables | Yes |  |  | Yes |  | Yes |  | Yes |  |

^a^ N/A: not applicable.

**Table S4.** Results for urban versus rural subgroups (n=6562).

| **Variable** | **Self-rated health** | | | |  | **Physical health** | | | |  | **Mental health** | | | |
| --- | --- | --- | --- | --- | --- | --- | --- | --- | --- | --- | --- | --- | --- | --- |
|  | **Urban** | | **Rural** | |  | **Urban** | | **Rural** | |  | **Urban** | | **Rural** | |
|  | **OR(95% CI)** | ***P* value** | **OR(95% CI)** | ***P* value** |  | **OR(95% CI)** | ***P* value** | **OR(95% CI)** | ***P* value** |  | **OR(95% CI)** | ***P* value** | **OR(95% CI)** | ***P* value** |
| **Internet use(ref:no)** |  |  |  |  |  |  |  |  |  |  |  |  |  |  |
| Yes | 1.37(1.34-1.65) | .001 | 1.67(1.45-1.93) | <.001 |  | 1.12(0.93-1.36) | .240 | 1.57(1.36-1.81) | <.001 |  | 1.20(0.99-1.45) | .064 | 1.40(1.22-1.62) | <.001 |
| **Gender (re: female)** |  |  |  |  |  |  |  |  |  |  |  |  |  |  |
| Male | 1.09(0.94-1.26) | .265 | 1.43(1.27-1.61) | <.001 |  | 1.08(0.93-1.25) | .340 | 1.42(1.26-1.60) | <.001 |  | 1.16(1.00-1.35) | .048 | 1.40(1.25-1.580) | <.001 |
| **Age** | 0.97(0.97-0.98) | <.001 | 0.98(0.97-0.99) | <.001 |  | 0.98(0.97-0.99) | <.001 | 0.98(0.97-0.99) | <.001 |  | 1.01(1.00-1.02) | .135 | 1.01(1.00-1.01) | .048 |
| **Marital status (ref:unmarried)** |  |  |  |  |  |  |  |  |  |  |  |  |  |  |
| Married | 1.14(0.93-1.40) | .208 | 1.08(0.92-1.27) | .333 |  | 1.28(1.04-1.57) | .018 | 1.11(0.95-1.30) | .198 |  | 1.28(1.04-1.57) | .020 | 1.28(1.09-1.50) | .003 |
| **Education (ref:illiterate)** |  |  |  |  |  |  |  |  |  |  |  |  |  |  |
| ≤Primary school | 1.06(0.73-1.54) | .775 | 1.03(0.89-1.20) | .677 |  | 1.02(0.72-1.46) | .895 | 1.03(0.88-1.19) | .744 |  | 1.26(0.89-1.80) | .195 | 1.03(0.89-1.20) | .701 |
| Middle school | 0.96(0.67-1.36) | .800 | 1.18(0.99-1.41) | .061 |  | 1.42(1.02-1.98) | .036 | 1.25(1.05-1.49) | .012 |  | 1.39(1.00-1.94) | .048 | 1.22(1.02-1.45) | .030 |
| High school | 1.29(0.90-1.85) | .160 | 1.24(0.98-1.56) | .080 |  | 1.89(1.35-2.65) | <.001 | 1.22(0.96-1.55) | .101 |  | 1.46(1.04-2.05) | .027 | 1.25(0.98-1.59) | .069 |
| ≥College | 1.32(0.90-1.93) | .161 | 1.42(0.89-2.28) | .145 |  | 1.91(1.33-2.76) | .001 | 1.40(0.86-2.27) | .180 |  | 1.36(0.94-1.96) | .100 | 1.23(0.77-1.97) | .380 |
| **Exercise(ref: no)** |  |  |  |  |  |  |  |  |  |  |  |  |  |  |
| Yes | 1.95(1.67-2.29) | <.001 | 1.39(1.23-1.57) | <.001 |  | 1.91(1.63-2.25) | <.001 | 1.34(1.18-1.51) | <.001 |  | 1.68(1.43-1.97) | <.001 | 1.20(1.06-1.36) | .003 |
| **Family size** | 0.97(0.91-1.04) | .389 | 1.04(0.99-1.09) | .071 |  | 1.03(0.96-1.10) | .438 | 1.03(0.99-1.08) | .167 |  | 1.03(0.96-1.10) | .471 | 1.02(0.98-1.06) | .380 |
| **Family income** | 1.10(1.04-1.15) | .001 | 1.08(1.05-1.11) | <.001 |  | 1.06(1.01-1.11) | .023 | 1.07(1.04-1.09) | <.001 |  | 1.05(0.99-1.10) | .085 | 1.05(1.02-1.07) | .001 |

**Table S5.** Results for eastern versus western subgroups (n=6562).

| **Variable** | **Self-rated health** | | | |  | **Physical health** | | | |  | **Mental health** | | | |
| --- | --- | --- | --- | --- | --- | --- | --- | --- | --- | --- | --- | --- | --- | --- |
|  | **Eastern** | | **Western** | |  | **Eastern** | | **Western** | |  | **Eastern** | | **Western** | |
|  | **OR(95% CI)** | ***P* value** | **OR(95% CI)** | ***P* value** |  | **OR(95% CI)** | ***P* value** | **OR(95% CI)** | ***P* value** |  | **OR(95% CI)** | ***P* value** | **OR(95% CI)** | ***P* value** |
| **Internet use(ref:no)** |  |  |  |  |  |  |  |  |  |  |  |  |  |  |
| Yes | 1.45(1.24-1.69) | <.001 | 2.03(1.60-2.57) | <.001 |  | 1.21(1.03-1.41) | .018 | 2.14(1.69-2.71) | <.001 |  | 1.40(1.20-1.64) | <.001 | 1.29(1.02-1.63) | .037 |
| **Gender (re: female)** |  |  |  |  |  |  |  |  |  |  |  |  |  |  |
| Male | 1.23(1.09- 1.39) | .001 | 1.49(1.22-1.81) | <.001 |  | 1.23(1.09-1.39) | .001 | 1.47(1.21-1.78) | <.001 |  | 1.26(1.11-1.43) | <.001 | 1.39(1.14-1.69) | .001 |
| **Age** | 0.98(0.97- 0.98) | <.001 | 0.98(0.97-0.99) | <.001 |  | 0.98(0.97-0.98) | <.001 | 0.98(0.97-0.99) | <.001 |  | 1.01(1.00-1.01) | .041 | 1.01(1.00-1.02) | .191 |
| **Marital status (ref:unmarried)** |  |  |  |  |  |  |  |  |  |  |  |  |  |  |
| Married | 1.11(0.94- 1.32) | .222 | 1.06(0.82-1.37) | .664 |  | 1.21(1.02-1.43) | .026 | 1.16(0.90-1.50) | .248 |  | 1.30(1.10-1.54) | .003 | 1.13(0.88-1.46) | .349 |
| **Education (ref:illiterate)** |  |  |  |  |  |  |  |  |  |  |  |  |  |  |
| ≤Primary school | 1.06(0.85- 1.33) | .618 | 1.04(0.82-1.34) | .730 |  | 1.01(0.81-1.25) | .959 | 1.06(0.83-1.35) | .642 |  | 0.92(0.74-1.14) | .463 | 1.18(0.92-1.51) | .194 |
| Middle school | 0.96(0.76- 1.20) | .695 | 1.22(0.91-1.65) | .183 |  | 1.34(1.07-1.67) | .010 | 1.15(0.86-1.55) | .347 |  | 1.05(0.84-1.31) | .685 | 1.17(0.87-1.57) | .302 |
| High school | 1.17(0.91- 1.51) | .218 | 1.78(1.20-2.63) | .004 |  | 1.51(1.18-1.93) | .001 | 1.37(0.93-2.00) | .110 |  | 1.05(0.82-1.34) | .720 | 1.59(1.07-2.36) | .020 |
| ≥College | 1.21(0.90- 1.62) | .200 | 1.48(0.77-2.87) | .244 |  | 1.65(1.24-2.22) | .001 | 1.14(0.60-2.19) | .691 |  | 0.93(0.70-1.25) | .640 | 2.30(1.17-4.52) | .016 |
| **Exercise(ref: no)** |  |  |  |  |  |  |  |  |  |  |  |  |  |  |
| Yes | 1.60(1.40- 1.82) | <.001 | 1.71(1.38-2.11) | <.001 |  | 1.61(1.41-1.84) | <.001 | 1.58(1.28-1.95) | <.001 |  | 1.43(1.26-1 .63) | <.001 | 1.21(0.98-1.49) | .084 |
| **Residency(ref:rural)** |  |  |  |  |  |  |  |  |  |  |  |  |  |  |
| Urban | 0.84(0.72- 0.98) | .029 | 0.89(0.66-1.19) | .431 |  | 1.03(0.88-1.21) | .699 | 0.87(0.65-1.16) | .340 |  | 1.10(0.94-1.28) | .244 | 1.00(0.75-1.33) | .978 |
| **Family size** | 1.02(0.97- 1.08) | .455 | 1.02(0.95-1.09) | .590 |  | 1.07(1.01-1.13) | .029 | 0.98(0.91-1.04) | .473 |  | 1.08(1.02-1.14) | .010 | 0.96(0.90-1.03) | .256 |
| **Family income** | 1.09(1.05- 1.12) | <.001 | 1.05(1.00-1.10) | .075 |  | 1.04(1.00-1.08) | .030 | 1.07(1.02-1.13) | .011 |  | 1.03(1.00-1.07) | .093 | 1.08(1.03-1.14) | .004 |

**Table S6.** Results for high-income versus low-income subgroups (n=6562).

| **Variable** | **Self-rated health** | | | |  | **Physical health** | | | |  | **Mental health** | | | |
| --- | --- | --- | --- | --- | --- | --- | --- | --- | --- | --- | --- | --- | --- | --- |
|  | **High-income** | | **Low-income** | |  | **High-income** | | **Low-income** | |  | **High-income** | | **Low-income** | |
|  | **OR(95% CI)** | ***P* value** | **OR(95% CI)** | ***P* value** |  | **OR(95% CI)** | ***P* value** | **OR(95% CI)** | ***P* value** |  | **OR(95% CI)** | ***P* value** | **OR(95% CI)** | ***P* value** |
| **Internet use(ref:no)** |  |  |  |  |  |  |  |  |  |  |  |  |  |  |
| Yes | 1.37(1.17-1.61) | <.001 | 1.74(1.47-2.05) | <.001 |  | 1.17(1.00-1.38) | .050 | 1.61(1.37- 1.90) | <.001 |  | 1.16(0.98- 1.36) | .080 | 1.49(1.26-1.76) | <.001 |
| **Gender (re: female)** |  |  |  |  |  |  |  |  |  |  |  |  |  |  |
| Male | 1.31(1.15-1.48) | <.001 | 1.30(1.13-1.49) | <.001 |  | 1.26(1.11-1.44) | <.001 | 1.26(1.11- 1.45) | .001 |  | 1.28(1.13- 1.45) | <.001 | 1.31(1.14- 1.50) | .020 |
| **Age** | 0.97(0.96-0.98) | <.001 | 0.99(0.98-0.99) | <.001 |  | 0.97(0.97-0.98) | <.001 | 0.98(0.98-0.99) | <.001 |  | 1.00(1.00-1.01) | .455 | 1.01(1.00- 1.02) | .001 |
| **Marital status (ref:unmarried)** |  |  |  |  |  |  |  |  |  |  |  |  |  |  |
| Married | 1.05(0.86-1.27) | .635 | 1.19(1.01-1.41) | .040 |  | 1.20(0.99-1.46) | .060 | 1.18(1.00-1.40) | .046 |  | 1.25(1.03- 1.52) | .024 | 1.31(1.11- 1.55) | <.001 |
| **Education (ref:illiterate)** |  |  |  |  |  |  |  |  |  |  |  |  |  |  |
| ≤Primary school | 0.93(0.73-1.19) | .575 | 1.14(0.96-1.35) | .130 |  | 0.94(0.74-1.19) | .061 | 1.12(0.95-1.32) | .180 |  | 1.10(0.86- 1.39) | .455 | 1.06(0.89- 1.25) | .516 |
| Middle school | 1.04(0.81-1.32) | .778 | 1.23(1.01-1.51) | .042 |  | 1.34(1.06-1.69) | .016 | 1.32(1.08-1.61) | .007 |  | 1.28(1.01- 1.62) | .042 | 1.25(1.02- 1.53) | .030 |
| High school | 1.30(1.00-1.69) | .049 | 1.25(0.95-1.64) | .118 |  | 1.60(1.24-2.06) | <.001 | 1.39(1.06- 1.83) | .019 |  | 1.37(1.07- 1.77) | .015 | 1.22(0.92- 1.61) | .159 |
| ≥College | 1.38(1.03-1.86) | .030 | 1.08(0.55-2.12) | .813 |  | 1.71(1.28-2.28) | <.001 | 1.51(0.75-3.05) | .251 |  | 1.27(0.95- 1.70) | .105 | 1.48(0.72-3.03) | .287 |
| **Exercise(ref: no)** |  |  |  |  |  |  |  |  |  |  |  |  |  |  |
| Yes | 1.71(1.50-1.95) | <.001 | 1.42(1.23-1.63) | <.001 |  | 1.61(1.41-1.85) | <.001 | 1.41(1.23- 1.62) | <.001 |  | 1.40(1.22- 1.60) | <.001 | 1.31(1.14- 1.51) | <.001 |
| **Residency(ref:rural)** |  |  |  |  |  |  |  |  |  |  |  |  |  |  |
| Urban | 0.76(0.65-0.89) | .001 | 1.04(0.85-1.27) | .721 |  | 0.92(0.78-1.07) | .281 | 1.21(0.99- 1.48) | .062 |  | 1.06(0.90- 1.23) | .491 | 1.19(0.97- 1.47) | .095 |
| **Family size** | 1.02(0.97-1.07) | .514 | 1.03(0.98-1.09) | .282 |  | 1.02(0.97-1.07) | .446 | 1.04(0.98- 1.10) | .188 |  | 1.03(0.98- 1.08) | .227 | 1.00(0.95- 1.06) | .964 |

**Table S7.** Results for high-education versus low-education subgroups (n=6562).

| **Variable** | **Self-rated health** | | | |  | **Physical health** | | | |  | **Mental health** | | | |
| --- | --- | --- | --- | --- | --- | --- | --- | --- | --- | --- | --- | --- | --- | --- |
|  | **High-education** | | **Low-education** | |  | **High-education** | | **Low-education** | |  | **High-education** | | **Low-education** | |
|  | **OR(95% CI)** | ***P* value** | **OR(95% CI)** | ***P* value** |  | **OR(95% CI)** | ***P* value** | **OR(95% CI)** | ***P* value** |  | **OR(95% CI)** | ***P* value** | **OR(95% CI)** | ***P* value** |
| **Internet use(ref:no)** |  |  |  |  |  |  |  |  |  |  |  |  |  |  |
| Yes | 1.56(1.34-1.80) | <.001 | 1.61(1.36-1.92) | <.001 |  | 1.49(1.28-1.73) | <.001 | 1.29(1.09- 1.53) | .004 |  | 1.35(1.17- 1.57) | <.001 | 1.27(1.07- 1.52) | .007 |
| **Gender (re: female)** |  |  |  |  |  |  |  |  |  |  |  |  |  |  |
| Male | 1.18(1.04-1.34) | .010 | 1.41(1.24-1.61) | <.001 |  | 1.18(1.04-1.34) | .011 | 1.37(1.20- 1.56) | <.001 |  | 1.19(1.05- 1.35) | .006 | 1.41(1.23- 1.61) | <.001 |
| **Age** | 0.97(0.97-0.98) | .164 | 0.98(0.98-0.99) | <.001 |  | 0.98(0.97-0.99) | <.001 | 0.98(0.97-0.99) | <.001 |  | 1.00(1.00-1.01) | .278 | 0.01(1.00- 1.01) | .058 |
| **Marital status (ref:unmarried)** |  |  |  |  |  |  |  |  |  |  |  |  |  |  |
| Married | 1.14(0.95-1.38) | <.001 | 1.06(0.90-1.25) | .484 |  | 1.18(0.98-1.42) | .086 | 1.15(0.97- 1.35) | .101 |  | 1.23(1.02- 1.49) | .031 | 1.30(1.10- 1.54) | .002 |
| **Exercise(ref: no)** |  |  |  |  |  |  |  |  |  |  |  |  |  |  |
| Yes | 1.64(1.44-1.87) | <.001 | 1.50(1.30-1.73) | <.001 |  | 1.53(1.34-1.75) | <.001 | 1.52(1.32- 1.76) | <.001 |  | 1.38(1.21- 1.58) | <.001 | 1.32(1.14- 1.52) | <.001 |
| **Residency(ref:rural)** |  |  |  |  |  |  |  |  |  |  |  |  |  |  |
| Urban | 0.87(0.75-1.02) | .082 | 0.89(0.73-1.09) | .255 |  | 1.11(0.96-1.30) | .166 | 0.95(0.78- 1.16) | .629 |  | 1.15(0.99- 1.34) | .063 | 1.02(0.84- 1.25) | .827 |
| **Family size** | 1.03(0.97-1.08) | .352 | 1.02(-0.97-1.07) | .506 |  | 1.02(0.96-1.07) | .566 | 1.05(0.99- 1.10) | .082 |  | 1.04(0.99- 1.09) | .152 | 1.01(0.96- 1.06) | .771 |
| **Family income** | 1.13(1.09-1.17) | <.001 | 1.06(1.03-1.10) | <.001 |  | 1.09(1.05-1.13) | <.001 | 1.04(1.01-0.08) | .005 |  | 1.05(0.02-0.09) | .009 | 1.04(1.01-0.07) | .023 |
